# The effect of macropore size of hydroxyapatite scaffold on the osteogenic differentiation of bone mesenchymal stem cells under perfusion culture

**DOI:** 10.1093/rb/rbab050

**Published:** 2021-09-07

**Authors:** Feng Shi, Dongqin Xiao, Chengdong Zhang, Wei Zhi, Yumei Liu, Jie Weng

**Affiliations:** 1 Collaboration Innovation Center for Tissue Repair Material Engineering Technology, College of Life Science, China West Normal University, No.1 Shida Road, Nanchong, Sichuan 637002, China; 2 Research Institute of Tissue Engineering and Stem Cells, Nanchong Central Hospital, the Second Clinical College of North Sichuan Medical College, No.97 Renmin South Road, Nanchong, Sichuan 637000, China; 3 College of Medicine, Southwest Jiaotong University, No.111 North 1st Section of Second Ring Road, Chengdu, Sichuan 610031, China; 4 College of Environmental Science and Engineering, China West Normal University, No.1 Shida Road, Nanchong, Sichuan 637002, China

**Keywords:** macropore size, HAp scaffolds, perfusion culture, osteogenic differentiation

## Abstract

Previous studies have proved that dynamic culture could facilitate nutrients transport and apply mechanical stimulation to the cells within three-dimensional scaffolds, thus enhancing the differentiation of stem cells towards the osteogenic phenotype. However, the effects of macropore size on osteogenic differentiation of stem cells under dynamic condition are still unclear. Therefore, the objective of this study was to investigate the effects of macropore size of hydroxyapatite (HAp) scaffolds on osteogenic differentiation of bone mesenchymal stem cells under static and perfusion culture conditions. *In vitro* cell culture results showed that cell proliferation, alkaline phosphate (ALP) activity, mRNA expression of ALP, collagen-I (Col-I), osteocalcin (OCN) and osteopontin (OPN) were enhanced when cultured under perfusion condition in comparison to static culture. Under perfusion culture condition, the ALP activity and the gene expression of ALP, Col-I, OCN and OPN were enhanced with the macropore size decreasing from 1300 to 800 µm. However, with the further decrease in macropore size from 800 to 500 µm, the osteogenic related gene expression and protein secretion were reduced. Computational fluid dynamics analysis showed that the distribution areas of medium- and high-speed flow increased with the decrease in macropore size, accompanied by the increase of the fluid shear stress within the scaffolds. These results confirm the effects of macropore size on fluid flow stimuli and cell differentiation, and also help optimize the macropore size of HAp scaffolds for bone tissue engineering.

## Introduction

Bone tissue engineering is a promising strategy for the repair of large bone defects, which is using scaffolds, cells and growth-stimulating bioactive factors to achieve biofunctional engineered tissue. Porous three-dimensional (3D) scaffolds not only enable the exchange of nutrition metabolism and vascular ingrowth but also provide a suitable environment for bone mesenchymal stem cells (BMSCs) migration, proliferation and differentiation to facilitate tissue regeneration. Calcium phosphate (CaP) ceramics (e.g. hydroxyapatite (HAp), tricalcium phosphate, their biphasic calcium phosphate (BCP) composites) have been widely used in bone repair due to their chemical similarity to human bone. The physical properties of CaP scaffolds, including micropore structure and macropore structure, have been shown to play key roles in bone regeneration *in vitro and in vivo*. The presence of micropore structure in HAp porous scaffold could induce new bone formation after ectopic implantation, whereas no bone formation was observed in HAp without micropore structure [1, 2]. Also, the macropore structures of CaP scaffolds, including interconnectivity, macropore size and macroporosity, play important roles in osteoinduction. Especially for macropore size, our previous study showed that HAp scaffold with macropore size of 750–900 μm exhibited the best performance on *in vivo* angiogenesis and osteogenesis compared with macropore size of 500–650 and 1100–1250 μm [3]. However, *in vitro* cell culture results showed that the osteogenic differentiation of BMSCs were not affected significantly by the macropore size, similar to previous studies [4, 5]. The inconsistent results between *in vitro* and *in vivo* are contributed to the different physiological microenvironment. For the traditional static cell culture, limited nutrients and metabolites can be exchanged in and out of scaffolds, leading to cell necrosis and limited extracellular matrix production within scaffolds [6, 7]. In this respect, dynamic culture, such as medium perfusion culture system, would provide a more physiological environment to cell growth.

Medium perfusion system can facilitate nutrients transport to the center of scaffolds and apply mechanical stimulation to cells within scaffolds, thus enhancing cellular osteogenesis via mechano-transduction signaling pathways [8, 9]. Studies have showed that mechanical stimulation via fluid shear stress within the scaffold was affected not only by the fluid parameters (e.g. flow rate, medium viscosity, temperature, etc.), but also by the macropore structure of the 3D scaffold [10, 11]. The macropore structure can influence the internal flow field distribution through the scaffold, thereby affecting cell biological response via mechanical stimuli [12]. However, few studies have been done to verify the effect of macropore structure, especially for macropore size, on the osteogenic differentiation of BMSCs under perfusion culture condition. Furthermore, the systematic relationship between flow field distribution affected by macropore size and cell differentiation has not been established.

**Figure 1.**
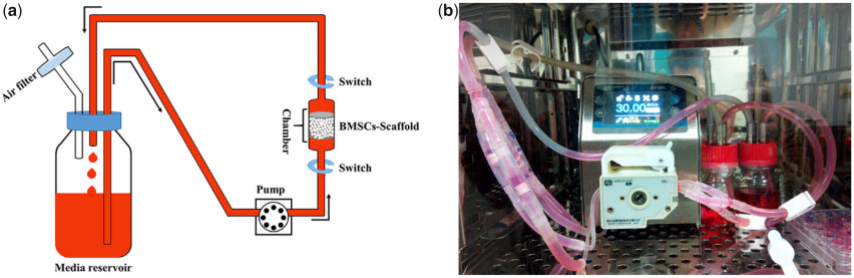
(a) Schematic diagram of the self-designed perfusion bioreactor (single circulation branch). Black arrow indicates the direction of culture medium perfusion. (b) Perfusion culture system in CO_2_ incubator.

Therefore, the aim of this study was to investigate the osteogenic differentiation of BMSCs in response to fluid flow within HAp scaffolds with different pore sizes. To accomplish this, BMSCs were seeded into three types of HAp scaffolds with various macropore sizes ranging from 500 to 1250 µm in a self-designed perfusion bioreactor (shown in Fig. 1). Cell viability, alkaline phosphate (ALP) activity and bone-related gene expressions (e.g. ALP, collagen-I (Col-I), osteocalcin (OCN) and osteopontin (OPN)) were measured and compared with those cultured statically. Furthermore, to explore the mechanism of macropore size on the osteogenic differentiation of cells under perfusion culture condition, a computational fluid dynamics (CFD) simulation was used in conjunction with micro-CT image of scaffolds to examine mechanical force changes within these scaffolds.

## Materials and methods

### Preparation of porous HAp scaffolds with various macropore sizes

Porous HAp scaffolds (diameter: 10 mm, height: 8 mm) with various macropore sizes in ranges of 500–650, 700–950 and 1100–1250 µm were fabricated using sugar spheres with various sizes according to our previous report [3]. Briefly, HAp powder was dispersed in LiCl/*N*,*N*-dimethylacetamide solution after chitin was completely dissolved. Sugar spheres were packed into cylindrical mold and formed templates after heat treatment at 70°C. Then, the HAp/chitin slurry was extruded into the sugar sphere templates and gelled by soaking into distilled water. After sugar spheres were completely dissolved in distilled water, the HAp/chitin samples were collected and dried at 80°C for 12 h. Finally, the samples were sintered at 500°C for 2 h to remove the chitin and further calcined at 1200°C for 2 h to obtain porous HAp scaffolds. The HAp scaffolds with various macropore sizes were defined as HAp-S (500–650 µm), HAp-M (700–950 µm) and HAp-L (1100–1250 µm). Scaffold morphology and macropore structure were characterized by scanning electron microscopy (SEM, FEI Quanta 200) and Super Nova PET/CT (SNPC-203). Also, the average diameters of macropore and interconnecting pore were measured from SEM images using Image J 1.49j software. For each scaffold group, 40 pores were randomly selected for statistical analysis (*n* = 3).

### Perfusion bioreactor setup

The self-designed perfusion bioreactor (Fig. 1) consisted of bioreactor chambers which could accommodate one cylindrical cellular scaffold with a diameter of 10 mm. A four-channel peristaltic pump was used to accurately control the flow rate of the culture medium to ensure the same perfusion rate in different chambers. Four perfusion chambers were connected in parallel into the same media flow circulating between medium reservoirs and culture chambers as indicated by black arrows, the medium was drawn out from the medium reservoir, pumped into the perfusion chamber, and returned to the same medium reservoir (Fig. 1a). Also, an air through hole in the medium storage bottle ensured the air exchange between the inside and outside of the medium reservoir. To check the sealing and sterility of the whole device, the perfusion bioreactor system was placed into a standard CO_2_ incubator at 37°C and 100 ml of Dulbecco’s Modified Eagle’s Medium with low glucose (L-DMEM) was loaded with circulation speed of 20 ml/min. During the continuous running process for 7 days, 2 ml of medium was removed every day for bacteriological cultivation (Fig. 1b).

### Seeding BMSCs into HAp scaffolds with various macropore sizes

BMSCs were obtained from bone marrow harvested from Sprague-Dawley rats (Sichuan Provincial Experimental Animal Center, Chengdu, Sichuan, China) according to our previous report [13]. BMSCs were seeded into the HAp scaffolds (HAp-S, HAp-M and HAp-L) with the assistance of semi-dynamic seeding device (as indicated in Supplementary Fig. S1). Briefly, 100 µl of cell suspension (5 × 10^5^ cells/per scaffold) was added into the scaffold by pushing and pulling the seeding device repeatedly. Then, the whole system was transferred into an incubator (37°C, 5% CO_2_) and incubated for 2 h to allow the cell attachment to the scaffolds. Then, the scaffolds were transferred into 24-well culture plates and 1.5 ml of growth culture medium (L-DMEM supplemented with 10% fetal bovine serum) was added to each well and further incubated for 3 days. Afterwards, cell/scaffold constructs were randomly divided into two groups: static culture group and perfusion culture group. For static culture group, the cell/scaffold constructs were continually cultured in the 24-well culture plates and the medium was replaced every 2 days. For the perfusion culture group, the cell/scaffold constructs were transferred into the bioreactor chamber and cultured with a flow rate at 30 μl/min for 6 h. Then, the cell/scaffold constructs were transferred into the 24-well plates and statically cultured for 6 h. This process was repeated twice a day for the perfusion culture group. For culture medium, growth culture medium was used for the cell proliferation, while osteogenic differentiation medium (growth culture medium supplemented with 10 mM β-glycerophosphate, 50 nM ascorbate-2-phosphate and 100 nM dexamethasone) was used to induce the osteogenic differentiation of BMSCs.

### Cell viability of BMSCs within HAp scaffolds with various macropore sizes

The proliferation of BMSCs in different groups was determined using Alamar Blue assays (AB, Invitrogen, Carlsbad, CA, USA) based on the metabolic activity of live cells, according to the manufacturer’ s protocol. At 1, 4 and 7 days, the cell/scaffold constructs were removed and washed with phosphate buffer saline (PBS), and 1.5 ml of AB solution (AB: L-DMEM = 1:9) were added and incubated at 37°C for 4 h. Then, 200 µl of the supernatant was pipetted into 96-well plate and read at 570 and 600 nm with a microplate reader (Molecular Devices, Sunnyvale, CA, USA).

The cell distribution within the porous scaffolds was observed by fluorescence staining using calcein-AM. After static/perfusion culture for 7 days, the scaffolds were washed with PBS and incubated in calcein-AM solution (5 μM) for 30 min. After washing with PBS, the scaffolds were transferred onto glass dish and scanned using an inverted confocal laser scanning microscope (CLSM; Nikon, A1, Tokyo, Japan) with scan range of 3.18×3.18×0.98 (length×width×height) mm. The captured images were reconstructed using NIS Viewer software for showing cell distribution in the porous scaffolds.

The cell apoptosis in different groups was determined using flow cytometry. After culture for 4 and 7 days, the cell/scaffold constructs were washed with PBS and digested using EDTA-free pancreatin. To ensure complete detachment of the cells inside the scaffolds, HAp scaffold was longitudinally splitted using a sterile surgical blade to expose internal cells. Then, the culture medium was added to terminate the digestion process. After centrifugation, the cells were washed twice with pre-cooled PBS at 4°C. The cells apoptosis in different groups was measured using the Annexin V-FITC/PI apoptosis detection kit according to the protocol.

### Osteogenic differentiation of BMSCs within HAp scaffolds with various macropore sizes

The ALP activity of the BMSCs was determined using a Quantichrom ALP assay kit (BioAssay systems, USA). After culture for 4, 7 and 14 days, the cell/scaffold construct was removed and washed with physiological saline, and transferred into 1.5 ml of EP tube. After adding 400 μl of cell lysate, the construct was crushed with a sterile glass rod in an ice box and further frozen and thawed three times. Then, 50 µl of the supernatant was mixed with 50 µl of ALP assay working solution, incubated at 37°C for 15 min and terminated using 25 µl of a 3 M NaOH solution. The absorbance value was measured at 405 nm. The lysate was used as a blank control. Meanwhile, the total protein concentration was detected using BCA protein assay kit (Beyotime Biotechnology, Inc., Shanghai, China). The ALP activity was expressed as the ALP absorbance value divided by the total protein concentration.

The expression of bone-related genes including ALP, Col-I, OCN and OPN was measured by a quantitative real-time polymerase chain reaction (RT-qPCR) system (CFX96, Bio-rad, USA). Total RNA was extracted from the BMSCs within the scaffolds after culture for 4, 7 and 14 days using TRIzol regent (Invitrogen Inc., Carlsbad, CA, USA) according to the manufacturer’s instructions [14]. The primer sequences for ALP, Col-I, OCN, OPN and glyceraldehyde-3-phosphate-dehydrogenase (GAPDH) are listed in Table 1. The relative expressions of bone-related genes were normalized according to the GAPDH expression.

**Table 1.** The primer sequence of qPCR

Gene	Genbank accession	Primer sequences (5′ to 3′)	Size (bp)
ALP	NM_013059.1	F: TACTCGGACAATGAGATGCGCC R: TTGTGCATTAGCTGATAGGCGA	74
Col-I	NM_053356.1	F: GCCAAGAATGCATACAGCCG R: GACACCCCTTCTGCGTTGTA	101
OCN	NM_013414.1	F: CCGTTTAGGGCATGTGTTGC R: TTTCGAGGCAGAGAGAGGGA	98
OPN	NM_001108550.1	F: TGGTGAGAGGAAGCAAGCAG R: GCTGAAGCGCTTATCTTGGC	113
GAPDH	NM_017008.4	F: GGACCAGGTTGTCTCCTGTG R: CATTGAGAGCAATGCCAGCC	80

### CFD simulation for evaluation of the flow field distribution within the scaffolds with various macropore sizes

Firstly, micro-CT measurement (µCT 80, Scanco, Switzerland) was used to reconstruct the 3D images of HAp scaffolds (diameter: 10 mm, height: 8 mm) with various macropore sizes. The micro-CT scan was performed layer-by-layer along with the height of the sample with a current of 800 mA and a voltage of 50 kV. The pixel size was 14.6 µm × 14.6 µm (with a stereo resolution of 14.6 µm). The images were reconstructed using supporting software (Mimics 10.0, Materialise Inc., Plymouth, MI, USA) to reconstruct 3D gray scale images. Then, the computer theoretical models of HAp scaffolds were established using Solid Works software, according to the parameters of macropore and interconnect structure of the actual scaffolds.

Secondly, to verify the influence of macropore size on the internal flow field within scaffolds, the theoretical formula calculation method and CFD simulation were incorporated to investigate the microfluidic flow field and shear stress within different macropore structures. To calculate the fluid shear stress within different macropore structures under the perfusion condition, the volume velocity of the perfusion fluid was converted to the average linear velocity through the macropores. Combined with the parameters of macropore structure and perfusion fluid, the average fluid shear stress on cells within the scaffolds was calculated according to (1) [15, 16]. Furthermore, during the CFD simulation, the medium was defined as an incompressible Newtonian fluid (density: 1030 kg/m^3^, viscosity: 0.025 Pa·s) and the linear velocity was based on the volume velocity of perfusion system (30 μl/min). The fluid outlet pressure was defined as 0 Pa, and the fluid flow mode was set as laminar flow mode.

(1) $$\tau_{W}=\frac{8\cdot \mu \cdot Q}{{\pi \Phi \left(D/2\right)}^{2}\overset{-}{d_{p}}}$$

where *µ* is the perfusion medium viscosity (37°C), $\overset{-}{d_{p}}$ is the average macropore size, *Q* is the perfusion fluid volume velocity, *Ф* is the scaffold porosity and *D* is the scaffold diameter.

### Statistical analysis

During the experiments, five samples per group were used for statistical analysis. Each experiment was repeated for three times. All the data were presented as mean ± SD. Differences between the groups were determined by Kruskal–Wallis one-way ANOVA on ranks followed by Mann–Whitney multiple comparison test (SPSS 20, IBM, Armonk, NY, USA). A *P* value <0.05 was considered significant difference, while a *P* value <0.01 was considered highly significant difference.

## Results

### Characterization of HAp scaffolds with various macropore sizes

**Figure 2.**
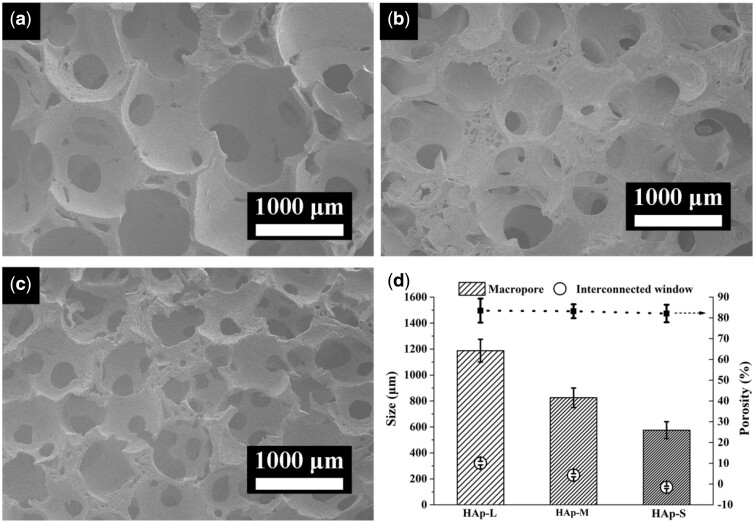
SEM images of porous HAp scaffolds with different macropore sizes: (a) HAp-L; (b) HAp-M; (c) HAp-S; and (d) pore structure parameters of different scaffolds.

The morphologies of HAp scaffolds with various macropore sizes were shown in Fig. 2. The prepared HAp scaffolds with macropores ranging from 1100–1250 µm (Fig. 2a, HAp-L), 700–950 µm (Fig. 2b, HAp-M) to 500–650 µm (Fig. 2c, HAp-S) all possessed excellent interconnectivity, which represented similar interconnecting structure on the macropore walls (Supplementary Fig. S3). The average interconnecting pores for HAp-L, HAp-M and HAp-S were 321 ± 13.9, 228 ± 12.1 and 135 ± 9.3 µm, respectively (Fig. 2d), keeping almost the same ratio (∼0.26) of interconnecting pores diameter to macropore diameter. Moreover, the three types of HAp scaffolds showed almost the same porosity of ∼83% (Fig. 2d).

### Cell proliferation, distribution and apoptosis within scaffolds

**Figure 3.**
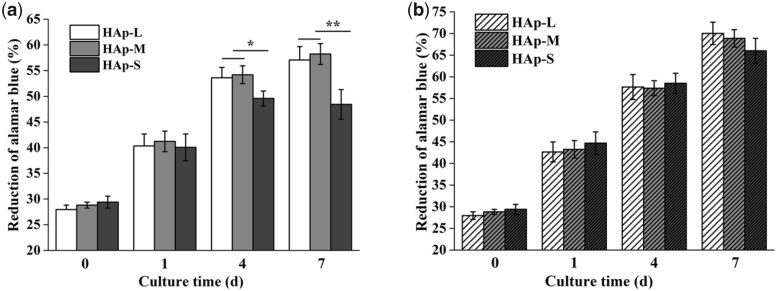
Cell proliferation in different HAp scaffolds under various culture conditions: (a) static culture and (b) perfusion culture.

The cell proliferation of BMSCs within HAp scaffolds with various macropores under static or perfusion culture condition was examined using Alamar Blue assays. As shown in Fig. 3, the cell number in all HAp scaffolds showed an increasing trend with the culture time in both culture conditions. For the perfusion culture group (Fig. 3b), the cell proliferation in the three types of scaffolds had no significant difference. However, for the static culture group (Fig. 3a), the cell proliferation in the HAp-S at Day 4 was significantly lower than those in the HAp-L and HAp-M (*P *<* *0.05). At Day 7, the cell number in the HAp-S was highly significantly lower than those in the HAp-L and HAp-M (*P *<* *0.01). In addition, the cell number in the HAp-S at Day 7 was not increased significantly compared with that at Day 4. Moreover, the overall cell number in the perfusion culture group at each day was significantly higher than that in the static culture group (*P *<* *0.05).

**Figure 4.**
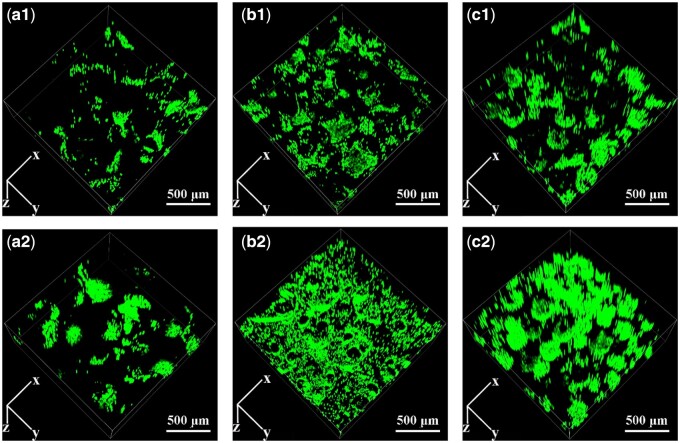
CLSM images of BMSCs attached on HAp scaffolds under (a1–c1) static culture condition and (a2–c2) perfusion culture condition: a1–a2: HAp-L, b1–b2: HAp-M, c1–c2: HAp-S.

The cell distribution of BMSCs within HAp scaffolds with various macropore sizes under static or perfusion culture condition was shown in Fig. 4. The fluorescence intensities of the cells within the three types of scaffolds in static culture group (a1–c1) were much weaker than those in the perfusion culture group (a2–c2), corresponding with the results of cell proliferation.

**Figure 5.**
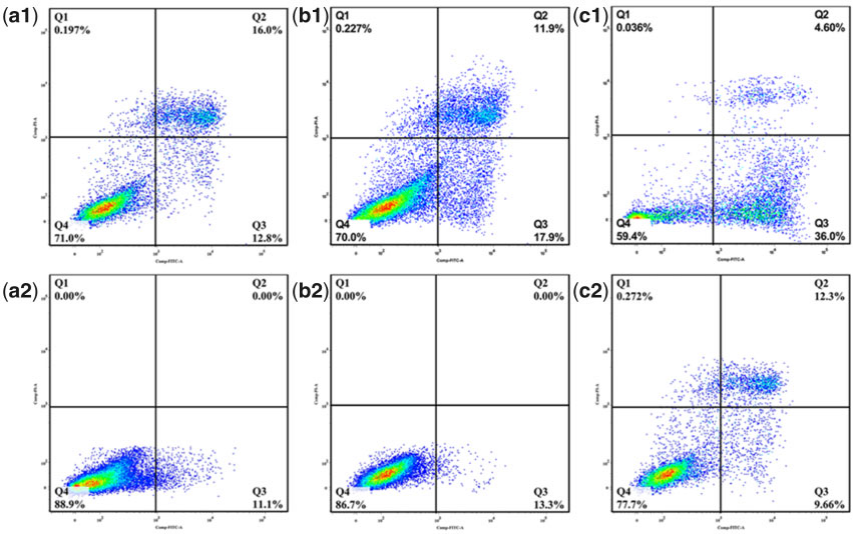
Apoptosis of BMSCs attached on HAp scaffolds under (a1–c1) static culture condition and (a2–c2) perfusion culture condition: a1–a2: HAp-L, b1–b2: HAp-M, c1–c2: HAp-S. Q1 represents necrotic cells; Q2 represents cells at a late stage of apoptosis; Q3 represents cells at an early stage of apoptosis; Q4 represents viable cells.

The cell apoptosis of BMSCs within HAp scaffolds with various macropore sizes under static or perfusion culture condition was shown in Fig. 5. Overall, the cell apoptosis ratio at early stage and late stage within the three types of scaffolds in the static culture group were much higher than those in the perfusion culture group. In addition, in the static culture group, the cell apoptosis ratio in HAp-S was the highest, which was consistent with the results of cell proliferation.

### Cell ALP activity and bone-related gene expression within scaffolds

**Figure 6.**
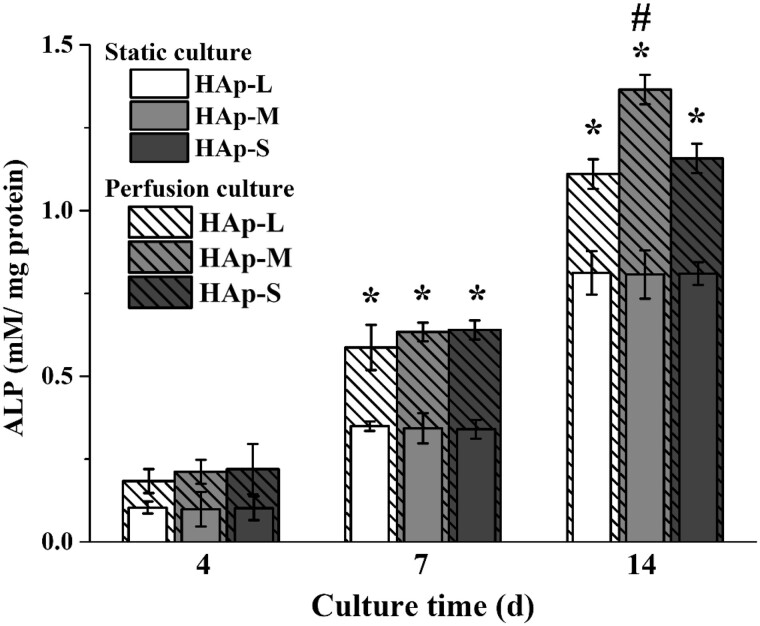
ALP activity of BMSCs on HAp scaffolds under static or perfusion culture. * indicates a statistically significant difference (*P *<* *0.05) between the static culture and perfusion culture. # indicates a statistically significant difference (*P *<* *0.05) among HAp-L, HAp-S and HAp-M in perfusion culture.

BMSCs differentiation along the osteogenic lineage within HAp scaffolds with various macropores under static or perfusion culture condition was examined using ALP activity tests (shown in Fig. 6). With the increase in culture time, the ALP activity of BMSCs within the three types of scaffolds in the static culture group or perfusion culture group was all enhanced. At Days 7 and 14, the overall ALP activity in the perfusion culture group was significantly higher than those in the static culture group. For the static culture group, there was no significant difference among the three types of scaffolds during the whole culture time. For the perfusion culture group, the ALP activity of BMSCs within HAp-M was significantly higher than those within HAp-S and HAp-L at Day 14.

**Figure 7.**
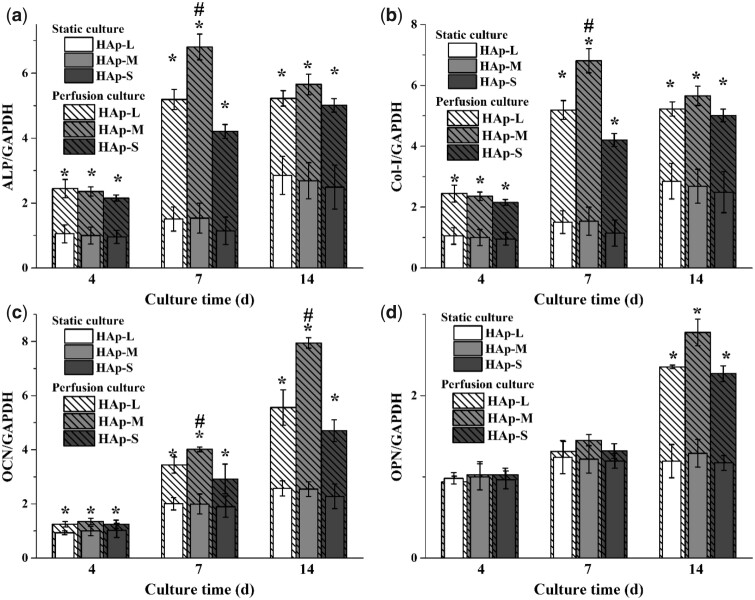
Expression of bone-related genes of BMSCs cultured on HAp scaffolds under static or perfusion culture. (a) ALP, (b) Col-I, (c) OCN and (d) OPN. * indicates a statistically significant difference (*P *<* *0.05) between the static culture and perfusion culture. # indicates a statistically significant difference (*P *<* *0.05) among HAp-L, HAp-S and HAp-M in perfusion culture.

Also, bone-related gene expressions of BMSCs within the three types of scaffolds under static or perfusion culture condition were evaluated using RT-qPCR (Fig. 7). During the whole culture time (at Days 4, 7 and 14), ALP, Col-I and OCN expressions on three types of scaffolds under perfusion culture condition were significantly higher than those under static culture condition. However, OPN expression on three types of scaffolds under perfusion culture condition was observed significantly higher than that under static condition only at Day 14. For static culture group, bone-relate genes, including ALP, Col-I, OCN and OPN, had no significant difference among the three types of scaffolds during the culture. For the perfusion culture group, ALP and Col-I expressions within HAp-M were significantly higher than those within HAp-S and HAp-L at Day 7, while OCN expression within HAp-M was significantly higher than those within HAp-S and HAp-L at Days 7 and 14.

### The flow field distribution and fluid shear stress within the scaffolds

**Figure 8.**
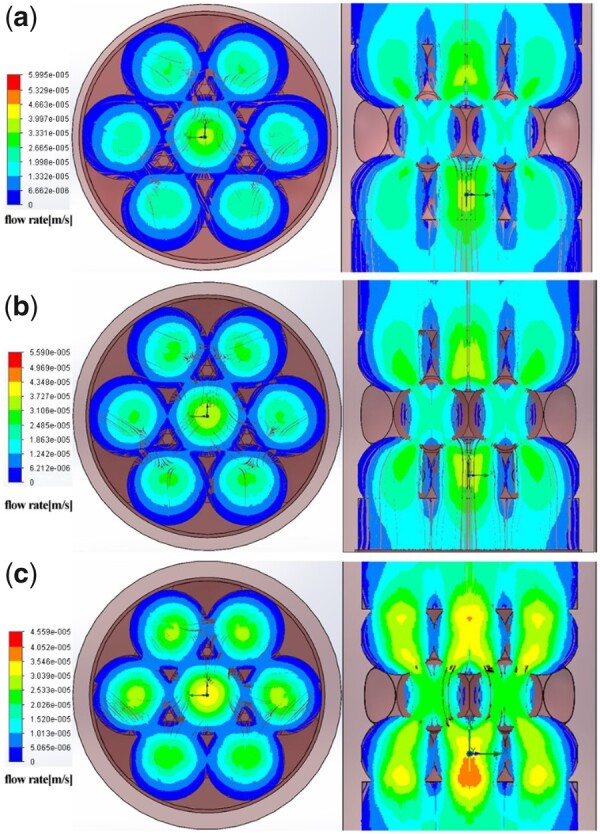
CFD fluid simulation analysis of the relationship between flow field distribution and macropore size. (a) HAp-L; (b) HAp-M; (c) HAp-S. The color bar shows the flow rate.

Firstly, the computer theoretical model of HAp scaffold was reconstructed based on the result of micro-CT scanning (shown in the Supplementary Fig. S2). CFD simulation (Fig. 8a) showed that when the interconnectivity ratios were consistent, the distribution trend of flow field within HAp scaffold was not affected by the macropore size. Medium- and high-velocity flow field were homogenously distributed around the interconnecting pores and decreased along the macropore wall for the three types of HAp scaffolds. However, the distribution area of medium- and high-velocity flow field decreased with the increased macropore size, indicating that the distribution area of medium- and high-velocity flow field showed a negative correlation with macropore size. Also, the maximum fluid velocity decreased with the increase in macropore size.

**Figure 9.**
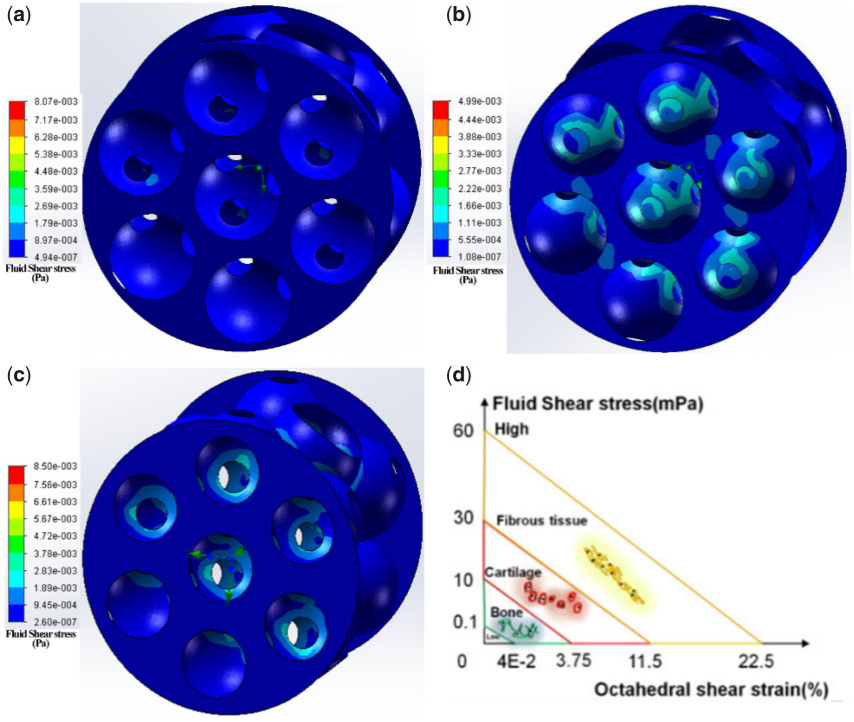
Fluid shear distribution (a–c) within different HAp scaffolds and (d) different tissues. (a) HAp-L; (b) HAp-M; (c) HAp-S. The color bar shows the shearing force.

Moreover, CFD simulation (Fig. 9 and Table 2) showed that the highest fluid shear stress was distributed around the interconnecting pores, and the radiation area of shear stress distribution was increased with the decrease in macropore size. Although the fluid velocity in the HAp-L center was the relatively high (8.07 MPa), the radiation area of shear stress distribution within scaffolds was very small, resulting in the lowest average shear stress (1.55 MPa). Moreover, the distribution area of high-velocity flow field within HAp-S was the largest, accompanied by highest average shear stress (5.78 MPa). Especially, the distribution range of shear stress within HAp-M was narrow and homogenous, with average value of 2.65 MPa. Although the average value of shear stress calculated by equation was lower than simulation result (Table 2), they all showed a similar trend that, under the same perfusion conditions, the average fluid shear stress within HAp scaffold had a negative correlation with the macropore size.

**Table 2.** Fluid shear force within different HAp scaffolds

Sample	CFD simulation			Calculation Average shear stress (MPa)
	Lowest shear stress (MPa)	Highest shear stress (MPa)	Average shear stress (MPa)	
HAp-L	4.94e−007	8.07	1.55	1.065
HAp-M	1.08e−007	4.99	2.65	1.530
HAp-S	2.6 e−007	8.50	5.78	2.224

## Discussion

In bone tissue engineering, the ideal macropore size for osteogenic activity in porous scaffolds is still controversial. Generally, the porous scaffolds with macropore size ranging from 100 to 1500 µm have been investigated [17–19]. It seems that macropore size <300 µm leads to osteochondral ossification while macropore size >300 µm encourages direct osteogenesis [18, 20, 21]. Especially, macropore size >300 µm is considered beneficial for cell migration, proliferation and blood vessel ingrowth [18, 20]. However, it seems that cell proliferation and ALP activity have no significant difference between scaffolds when macropore size is more than 500 µm [3, 5]. In addition, scaffolds with macropore size of 400 μm were preferred when the macropore size ranging from 75 to 900 μm were evaluated by cell culture [22]. Contrary to *in vitro* results, HAp scaffolds with macropore size of 800 µm yielded more bone than those with macropore size of 400 µm in goats [23]. Also, in our work, HAp scaffolds with macropore size of 700–950 µm showed better performance in guiding bone formation *in vivo*, compared with macropore size of 500–650 µm [3]. The different osteogenic effects of macropore size between *in vitro* and *in vivo* may be attributed to the different physiological microenvironment that cell located. The traditional static culture underestimates the important effects of sustained mass exchanges and biomechanical stimulus on bone remodeling. To some extent, perfusion culture can mitigate these undesired effects. Therefore, cell proliferation and differentiation behaviors within HAp scaffolds with different macropores were investigated using perfusion culture system in this study.

Compared with static culture, dynamic culture plays key roles in supporting the cultivation of large bone grafts *in vitro*. First, dynamic culture could enhance the mass transport of nutrients and metabolism in the scaffolds and overcome the diffusion limitation of static culture, especially for the central region of the scaffolds [24, 25]. Second, fluid flow in dynamic culture generates shear stress acting upon the seeding cells on the scaffolds and further enhances osteogenic differentiation and matrix production [8, 26–28]. Nowadays, bioreactors, such as spinner flasks, perfusion systems and rotating wall vessels, are usually used for achieving dynamic culture. As for spinner flasks and rotating wall vessels, it is difficult to control the internal fluid flow and shear stress acting on the scaffolds [29, 30]. In comparison, the fluid shear stress can be controlled by regulating the flow rate via a pump in the perfusion system. Also, perfusion bioreactors are designed based on the fluid flow completely passing through the whole scaffolds, instead of being around the surfaces [29, 12]. Therefore, a self-designed perfusion system (Fig. 1) was used for investigating the effects of macropore size on the cellular osteogenic differentiation under dynamic culture condition. The perfusion device designed in this study showed good sealing ability and maintained sterility after long-term operation. Also, the perfusion culture system established in this study used a pipeline branch system, which could apply for different sample sizes by changing the number of branches and branch lengths.

In perfusion culture system, the effect of fluid shear stress acting upon cells also depends on stimulating duration or action frequency [31, 32]. Compared with continuous perfusion, intermittent perfusion stimulation could enhance osteoblast differentiation by inducing Runx-2 expression [31]. Guignandon *et al*. [33] proposed that the continuous mechanical stimulation might weaken the effects of mechanical stimulation on cellular osteogenic differentiation due to cells adaptation to the mechanical environment after long-duration stimulation. Therefore, in this study, to investigate the effect of macropore size on the cellular behavior under perfusion condition, perfusion velocity was fixed at 30 μl/min and duration was 6 h before static culture in plates for 6 h. Under perfusion culture condition, the cell proliferation was enhanced but without no significant difference within the three types of scaffolds (HAp-L, HAp-M, HAp-S; Fig. 3). However, under static culture condition, the cell proliferation in HAp-S was much lower than those in the HAp-L and HAp-M at Days 4 and 7. This was probably because in the stationary medium, the transmission of nutrients and metabolic wastes was mainly dependent on physical diffusion. Compared with HAp-L and HAp-M, the smaller macropore size of HAp-S restricted the diffusion of substances into the scaffolds. The local nutrient consumption and the accumulation of metabolic wastes resulted in the inhibition of cell activity and proliferation ability [34]. The result was consistent with the cell fluorescent image, which showed that more viable cells were observed in HAp-S for the perfusion culture group (Fig. 4). Under dynamic culture condition, the flow medium was perfused through the whole scaffold and diffusional attenuation was avoided, thereby improving cell proliferation [35]. However, under perfusion condition, the early cell apoptosis rate in HAp-S was much higher than those in HAp-L and HAp-M. According to the formula (Table 2), the average shear stress was found to be inversely proportional to macropore size and the shear stress in HAp-S was highest. Thus, the maximum shear stress in HAp-S may contribute to the local cell apoptosis, similar to previous report of shear stress injury-induced BMSCs death [36].

Moreover, with prolonged time, cell ALP activity and bone-related gene expressions were significantly enhanced under perfusion condition, especially for cells within HAp-M. However, under static culture condition, cell ALP activity and gene expressions showed no significant difference among the three types of scaffolds. Under static culture condition, cells within the three types of scaffolds were induced into osteogenic differentiation only by the chemicals in the osteogenic differentiation medium [37]. Under perfusion culture condition, cell differentiation is not only determined by the chemical factors, but also by the fluid shear stress generated within scaffolds [38–40]. The synergistic effects between chemical factors and fluid shear stress resulted in the enhancement of osteogenic differentiation of BMSCs in the perfusion culture group. In addition, due to the difference in macropore size, BMSCs in scaffolds were subjected to different shear stress, which was inversely proportional to macropore size (Table 2). Studies have shown that the maximum shear stress that animal-derived cells could endure ranges between l and 5 Pa and the shear stress to induce differentiation within different tissues varied (Fig. 9d) [41, 42]. When the shear stress range was between 0.1 and 10 MPa, the bone tissue differentiation occurred. In our study, the cells within HAp-M showed the highest ALP activity and bone-related gene expression, suggesting that shear stress generated in HAp-M might be the most appropriate for promoting cell osteogenic differentiation.

Studies have demonstrated that the macropore structure (pore architectures, porosity and size) can change the flow field distribution during the flow field perfusion process, which further affected the nutrient transport and fluid shear stress distribution and finally affected the cell behaviors (e.g. attachment, distribution and proliferation) [11, 43, 44]. Appropriate shear stress may enhance cell differentiation and mineral deposition; however, too high shear stress may cause cell detachment and deformation [11, 44]. In this study, the flow perfusion rate and media viscosity were constant, so the variation of fluid shear stress was only caused by the macropore size of HAp scaffold. The average shear stress within different macropore sizes was obtained based on the formula (Table 2), since the direct measurement of distribution of fluid shear stress with a scaffold is not feasible. Thus, analytical predictions are used to estimate the fluid shear stress within a scaffold. For example, to study the effect of scaffold geometry on wall shear stress, Zhao *et al*. [43] applied a fluid–structure interaction computational model and found that scaffold pore size had a more important influence on mechanical stimulation within scaffolds compared with pore architecture and porosity. Here, we employed a CFD simulation combined with experimental approach to investigate the effect of macropore size on the flow field distribution and fluid shear stress within the scaffolds, and further evaluate the relationship between shear stress and cell differentiation. From the simulation results (Fig. 8), it was found that the maximum flow velocity within HAp scaffold decreased with the increase of the macropore size, and the distribution areas of the medium- and high-speed flow field within the scaffold also decreased with the increasing macropore size. The flow rate within HAp-M was slightly higher than that within HAp-L, but lower than that within HAp-S. Also, the distribution areas of medium and high velocity flow within HAp-M were significantly larger than those within HAp-L, but smaller than those within HAp-S. Due to the distribution difference in flow field and flow velocity within scaffolds, the shear stress on the macropore wall surface varied, which was decreased with the increasing macropore size (Table 2 and Fig. 9). Comparing the shear stress results between the simulation and formula calculation, they both showed the same trend that the average shear stress within HAp scaffold was negatively correlated with the macropore size. However, the value differences between the simulation and formula calculation are probably attributed to the ignorance of pore structure (e.g. interconnect pore) within scaffolds during the formula calculation. Compared with formula calculation, the fluid simulation can not only reflect the shear stress value, but also show the cloud distribution of internal shear stress.

**Figure 10.**
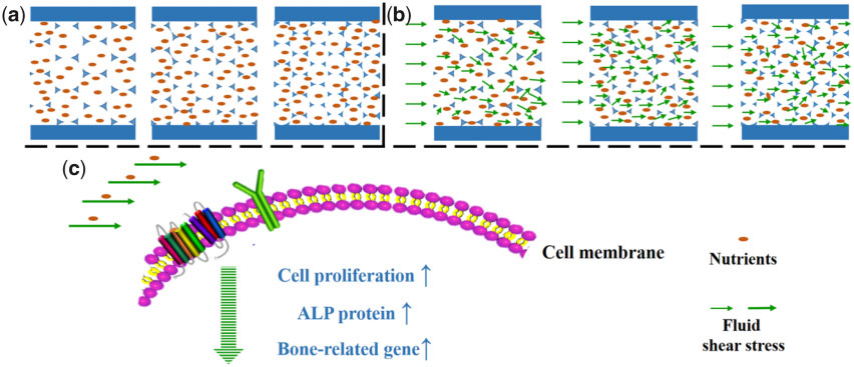
The proposed schematic diagram of the effect of macropore on cell behavior under static/perfusion culture condition. (a) Under static culture condition, nutrition distribution within scaffolds with different macropore sizes, (b) under perfusion culture condition, nutrient and fluid shear stress distribution within scaffolds with different macropore sizes.

Based on cell culture results and fluid shear stress analysis, the mechanism of macropore size-induced osteogenic differentiation of BMSCs under perfusion condition was proposed as follows (Fig. 10). Under static culture condition, limited nutrients and metabolites exchanged depending on physical diffusion. With the decrease in macropore size, the restricted effect of diffusion of substances became more obvious, resulting in the inhibition of cell proliferation. Under perfusion culture condition, appropriate perfusion velocity not only accelerates nutrients and metabolism exchange but also generates shear stress which could promote cell proliferation and osteogenic differentiation. The fluid shear stress acting upon the cells is affected not only by the fluidic parameters (e.g. flow velocity, medium viscosity, pulse frequency, etc.) but also by the macropore structure of the porous scaffold. With the increase in macropore size, the average fluid shear force decreased. However, the scaffold with macropore size of 750–900 μm showed the best performance in promoting cellular osteogenic differentiation compared with macropore size of 500–650 and 1100–1250 μm. The results suggested that cell differentiation would be maximally enhanced at appropriate fluid shear stress. Here, fluid shear stress of 2.65 MPa generated within HAp-M was more fit for cell osteogenic differentiation compared with 1.55 MPa generated within HAp-L and 5.78 MPa generated within HAp-S. Furthermore, the cell surface receptors (e.g. β1 integrins) would sense the stimulation of fluid shear stress, and further regulate cellular osteogenesis via initiating dynamic cascade of signaling pathways [8]. In this study, the mechano-stimulation intensity depends on the macropore structure of HAp scaffolds, indicating the key roles of macropore size in the bone healing process. Although the macropore size-induced osteogenic differentiation under perfusion condition is still not clear, our results do suggest that the macropore size plays crucial roles in the osteogenic differentiation of BMSCs in the dynamic culture environment.

## Conclusion

In this study, the influence of the macropore size of HAp scaffolds on BMSCs osteogenic differentiation was investigated under perfusion condition. The experiments revealed that the perfusion culture could significantly enhance the cell proliferation, ALP activity and bone-related gene expression as compared with static culture. Moreover, the osteogenic differentiation capacity of BMSCs within HAp-M was significantly stronger than those within HAp-S and HAp-L under perfusion culture condition. Theoretical calculation and CFD stimulation analysis showed that the average fluid shear stress within HAp scaffold had a negative correlation with the macropore size. In conclusion, perfusion culture not only overcomes the transport limitations of nutrition and waste under static culture, but also generates shear stress acting on cell growth. In addition, the fluid flow distribution and shear stress could be adjusted by the macropore size of HAp scaffolds and appropriate fluid shear stress generated within scaffolds has important effects on osteogenic differentiation of BMSCs. Therefore, *in vitro* perfusion culture could help guide a better understanding of macropore size-induced osteogenesis and optimize macropore size of tissue engineering scaffold.

## Supplementary data

Supplementary data are available at *REGBIO* online.

## Supplementary Material

rbab050_Supplementary_Data
