# Supplementary material for: The effect of macropore size of hydroxyapatite scaffold on the osteogenic differentiation of bone mesenchymal stem cells under perfusion culture
Source: Regen Biomater. 2021 Sep 7;8(6):rbab050. doi: 10.1093/rb/rbab050 (PMC8457200; doi:10.1093/rb/rbab050)

**Fig. S1** Schematic illustration of semi-dynamic seeding device. The black arrow indicates the flow direction of the cell suspension


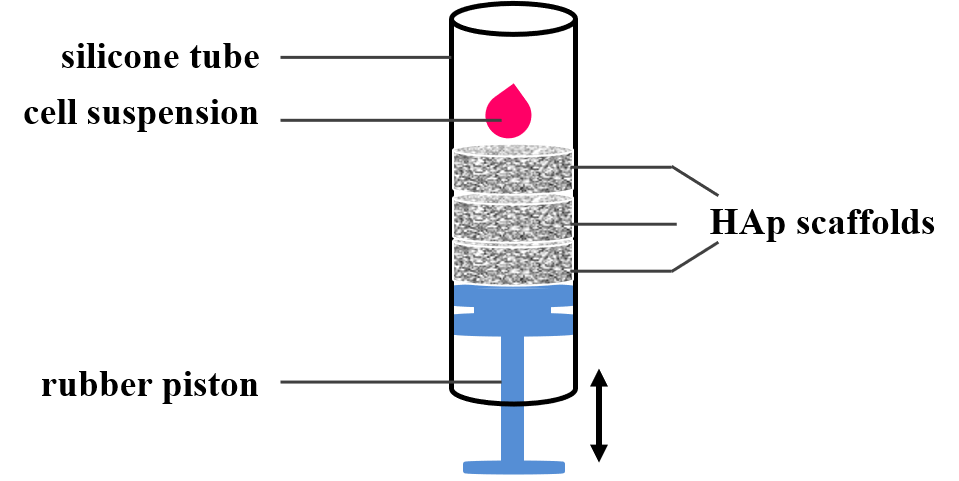


**Fig. S2** The model of 3D porous HAp scaffold: (a) the model of micro-CT scan reconstruction and (b) the model of theoretical calculation


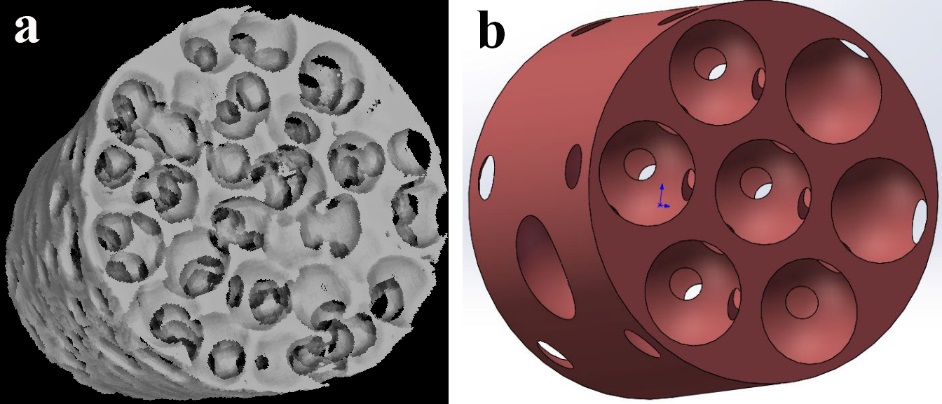


**Fig. S3** The porous structure of CT images of HAp scaffolds with different macropore sizes: the general view of (a1) HAp-L, (b1) HAp-M, (c1) HAp-S and the longitudinal section of (a2) HAp-L, (b2) HAp-M, (c2) HAp-S


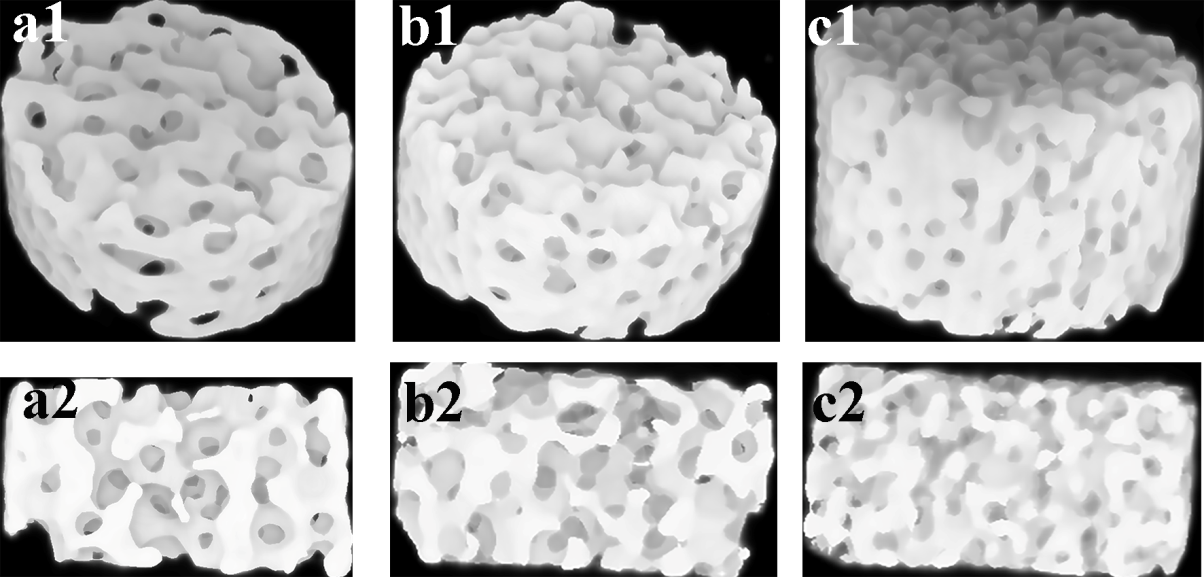

Supplement: rbab050_Supplementary_Data [file rbab050_supplementary_data.docx]
